# Consensus definition and diagnostic criteria for neonatal encephalopathy—study protocol for a real-time modified delphi study

**DOI:** 10.1038/s41390-024-03303-3

**Published:** 2024-06-20

**Authors:** Aoife Branagan, Tim Hurley, Fiona Quirke, Declan Devane, Petek E. Taneri, Nadia Badawi, Bharati Sinha, Cynthia Bearer, Frank H. Bloomfield, Sonia L. Bonifacio, Geraldine Boylan, Suzann K. Campbell, Lina Chalak, Mary D’Alton, Linda S. deVries, Mohamed El Dib, Donna M. Ferriero, Chris Gale, Pierre Gressens, Alistair J. Gunn, Sarah Kay, Beccy Maeso, Sarah B. Mulkey, Deirdre M. Murray, Karin B. Nelson, Tetyana H. Nesterenko, Betsy Pilon, Nicola J. Robertson, Karen Walker, Courtney J. Wusthoff, Eleanor J. Molloy, Steering Group for DEFiNE (Definition of Neonatal Encephalopathy), Aoife Branagan, Tim Hurley, Fiona Quirke, Declan Devane, Petek E. Taneri, Nadia Badawi, Cynthia Bearer, Frank H. Bloomfield, Sonia L. Bonifacio, Geraldine Boylan, Suzann K. Campbell, Lina Chalak, Mary D’Alton, Linda S. deVries, Mohamed El Dib, Donna M. Ferriero, Chris Gale, Pierre Gressens, Alistair J. Gunn, Sarah Kay, Deirdre M. Murray, Karin B. Nelson, Betsy Pilon, Nicola J. Robertson, Karen Walker, Courtney J. Wusthoff, Eleanor J. Molloy

**Affiliations:** 1 Discipline of Paediatrics, Trinity College Dublin, the University of Dublin, Dublin, Ireland; 2 Trinity Translational Medicine Institute (TTMI), St James Hospital & Trinity Research in Childhood Centre (TRiCC), Dublin, Ireland; 3 Neonatology, The Coombe Hospital, Dublin, Ireland; 4 Health Research Board Neonatal Encephalopathy PhD Training Network (NEPTuNE), Dublin, Ireland; 5 Health Research Board–Trials Methodology, Research Network (HRB-TMRN), University of Galway, Galway, Ireland; 6 School of Nursing and Midwifery, University of Galway, Galway, Ireland; 7 Evidence Synthesis Ireland, University of Galway, Galway, Ireland; 8 Cochrane Ireland, University of Galway, Galway, Ireland; 9 Cerebral Palsy Alliance Research Institute, Specialty of Child & Adolescent Health, Sydney Medical School, Faculty of Medicine & Health, The University of Sydney, Sydney, NSW Australia; 10 Grace Centre for Newborn Intensive Care, Sydney Children’s Hospital Network, The University of Sydney, Westmead, NSW Australia; 11 Department of Pediatrics, Boston Medical Center, Boston, MA USA; 12 Division of Neonatology, Department of Pediatrics, Rainbow Babies & Children’s Hospital, Cleveland, OH USA; 13 Case Western Reserve University School of Medicine, Cleveland, OH USA; 14 Liggins Institute, University of Auckland, Auckland, New Zealand; 15 Division of Neonatal and Developmental Medicine, Department of Pediatrics, Stanford University School of Medicine, Palo Alto, CA USA; 16 INFANT Research Centre, Cork, Ireland; 17 Department of Pediatrics and Child Health, University College Cork, Cork, Ireland; 18 Department of Physical Therapy, College of Applied Health Sciences, University of Illinois at Chicago, Chicago, IL USA; 19 Division of Neonatal-Perinatal Medicine, University of Texas Southwestern Medical Center, Dallas, TX USA; 20 Department of Obstetrics and Gynecology, Columbia University, New York, NY USA; 21 Department of Neonatology, Wilhelmina Children’s Hospital, University Medical Center Utrecht, Utrecht, the Netherlands; 22 Division of Newborn Medicine, Department of Pediatrics, Brigham and Women’s Hospital, Harvard Medical School, Boston, MA USA; 23 Departments of Neurology and Pediatrics, Weill Institute for Neurosciences, University of California San Francisco, San Francisco, CA USA; 24 Neonatal Medicine, School of Public Health, Faculty of Medicine, Chelsea and Westminster Campus, Imperial College London, London, UK; 25 Université Paris Cité, Inserm, NeuroDiderot, 75019 Paris, France; 26 Departments of Physiology and Paediatrics, School of Medical Sciences, University of Auckland, Auckland, New Zealand; 27 PEEPS-HIE, Manchester, UK; 28 James Lind Alliance, School of Healthcare Enterprise and Innovation, University of Southampton, Southampton, UK; 29 Children’s National Hospital, Washington, DC USA; 30 Departments of Neurology and Pediatrics, The George Washington University School of Medicine and Health Sciences, Washington, DC USA; 31 National Institutes of Health, National Institute of Neurological Diseases and Stroke, Bethesda, MD USA; 32 Department of Neonatology, Cleveland Clinic Children’s Hospital, Cleveland, OH USA; 33 Hope for HIE, West Bloomfield, MI USA; 34 Institute for Women’s Health, University College London, London, UK; 35 Centre for Clinical Brain Sciences, University of Edinburgh, Edinburgh, UK; 36 Department of Newborn Care, Royal Prince Alfred Hospital, Sydney, Local Health District, Sydney, Australia; 37 Faculty of Medicine and Health, University of Sydney, Sydney, Australia; 38 Division of Child Neurology, Stanford University, Palo Alto, CA USA; 39 Neonatology, Children’s Health Ireland, Dublin, Ireland; 40 Neurodisability, Children’s Hospital Ireland (CHI) at Tallaght, Dublin, Ireland

## Abstract

**Background:**

‘Neonatal encephalopathy’ (NE) describes a group of conditions in term infants presenting in the earliest days after birth with disturbed neurological function of cerebral origin. NE is aetiologically heterogenous; one cause is peripartum hypoxic ischaemia. Lack of uniformity in the terminology used to describe NE and its diagnostic criteria creates difficulty in the design and interpretation of research and complicates communication with families. The DEFINE study aims to use a modified Delphi approach to form a consensus definition for NE, and diagnostic criteria.

**Methods:**

Directed by an international steering group, we will conduct a systematic review of the literature to assess the terminology used in trials of NE, and with their guidance perform an online Real-time Delphi survey to develop a consensus diagnosis and criteria for NE. A consensus meeting will be held to agree on the final terminology and criteria, and the outcome disseminated widely.

**Discussion:**

A clear and consistent consensus-based definition of NE and criteria for its diagnosis, achieved by use of a modified Delphi technique, will enable more comparability of research results and improved communication among professionals and with families.

**Impact:**

- The terms Neonatal Encephalopathy and Hypoxic Ischaemic Encephalopathy tend to be used interchangeably in the literature to describe a term newborn with signs of encephalopathy at birth. This creates difficulty in communication with families and carers, and between medical professionals and researchers, as well as creating difficulty with performance of research.
- The DEFINE project will use a Real-time Delphi approach to create a consensus definition for the term ‘Neonatal Encephalopathy’.
- A definition formed by this consensus approach will be accepted and utilised by the neonatal community to improve research, outcomes, and parental experience.

## Introduction

‘Neonatal Encephalopathy’ (NE) refers to disturbance of neurological function of cerebral origin in a newborn born at, or near, term gestation.^1^ The American Academy of Paediatrics has defined NE as a ‘clinical syndrome of disturbed neurologic function in the earliest days after birth in an infant born at or beyond 35 weeks of gestation, manifested by a subnormal level of consciousness or seizures, often accompanied by difficulty with initiating and maintaining respiration, and depression of tone and reflexes’.^2^ Infants with NE often have difficulty initiating and sustaining respiration due to a central cause. Poor feeding due to difficulty in sucking and swallowing, or due to depressed consciousness, may be present. The defining characteristic of an encephalopathy is a disturbance of consciousness, which is enough alone to make a diagnosis of encephalopathy. The occurrence of seizures in the neonate, whether or not there are interictal neurologic abnormalities, may also be sufficient for a diagnosis of NE. Hypoxic ischaemic encephalopathy (HIE) is the subgroup of NE in which the aetiology of the NE is thought to be a decreased supply of oxygen in the peripartum period. The documentation of a sentinel event of birth severe enough to induce hypoxia, in an infant with NE, such as major placental abruption, cord accident or uterine rupture supports a diagnosis of HIE.

In the case of an asphyxial aetiology underlying a presentation of NE the only effective interventional therapy currently in use is therapeutic hypothermia (TH), which involves cooling of the newborn’s body temperature to 33.5 ± 0.5 degrees Celsius for 72 h to protect the brain from secondary injury. TH has been shown to reduce the risk of death or major neurodevelopmental disability at 18 months of age with a number needed to treat of seven.^3^ It is important to note that between 14 and 29% of infants in the control arm of the large clinical trials contributing to this meta-analysis experienced hyperthermia^4–6^ which has been shown to worsen the adverse outcomes seen in NE.^7,8^ Therefore, the actual neuroprotection offered by TH may be lower that this estimate suggests. The benefit of TH however, may be limited to infants in high income countries, which only experience about 10% of the global burden of NE. The benefit of TH in low-middle income countries is now less clear following the results of the HELIX trial, the largest trial of TH in a low-middle income setting, which recruited 408 infants in India, Sri Lanka and Bangladesh, and found that TH did not reduce the combined primary outcome of death or neurodisability at 2 years of age (risk ratio 1·06, 95% confidence interval 0·87–1·30, p = 0·55), and, indeed, actually increased death alone.^9^ Hypothermic treatment is most effective when initiated within 6 h of birth.^3^ Thus, the clinical diagnosis of NE due to a likely hypoxic cause must be made rapidly after birth in order to enable immediate decisions concerning treatment. Clinical trials assessing interventions for infants with NE with a presumed hypoxic aetiology have used a multi-step approach to identify these infants: First assessing for a specified Apgar score (e.g. <5 at 10 min) OR need for continued resuscitation at 10 min OR evidence of acidosis at birth or within 1 h of birth. This was then followed by a second step of assessing for moderate to severe encephalopathy on neurological examination AND/OR aEEG evidence of encephalopathy. The HEAL study has recently reported that using a very similar approach that 5% of babies had additional neurological diagnoses that probably contributed to encephalopathy and to adverse long-term neurodevelopmental outcomes, in addition to exposure to hypoxia-ischaemia.^10,11^

**Fig. 1:**
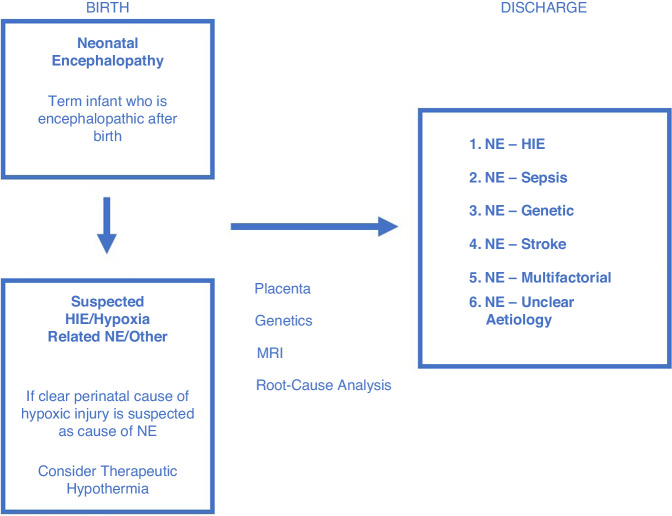
Neonatal Encephalopathy as an umbrella diagnosis. A scheme representing movement from the initial umbrella diagnosis of ‘Neonatal Encephalopathy’ to a final aetiological diagnosis after appropriate investigation and analysis is carried out.

Non-asphyxial causes of NE include metabolic and genetic disorders, congenital neuromuscular disorders, infection or sepsis, and neonatal stroke.^1,12^ The ultimate diagnosis will depend on the results of physical examination, expert examination of the placenta, genetic and metabolic investigations, investigation for sepsis or meningitis where indicated, neuro-imaging as well as a thorough investigation of family history (of congenital malformations, neurologic or medical disorders), pre-conceptional, antepartum events, obstetric course, delivery, and postnatal events^12^ (Fig. 1). A proportion of infants with NE will be found to have one of a number of other diagnoses - congenital malformations, abnormal genetics/genomics, or known syndromes and stroke among many others. Infants in whom aetiology is thought to be secondary to a specific non-asphyxial conditions must be identified separately for clinical trials, targeting these individual conditions. Even within the subgroup diagnosis of HIE, there is heterogeneity seen, ranging from severe acute hypoxia-ischaemia from an acute cause to subacute, intermittent hypoxemia of the course of labour and the chronic hypoxemia of pregnancy that is most often related to placental issues and of course, combinations such as acute on chronic hypoxemia, among other causes.

The aetiology of NE is in many cases complex and multifactorial, and despite thorough investigation the cause often remains undetermined^13^ (Fig. 1). The frequency of finding an identifiable non-asphyxial cause in NE is related to the availability of specific diagnostic tools and the effort and acumen with which differential diagnosis is pursued.^14^

The terms employed in neonatal brain injury: NE, HIE and perinatal asphyxia (PA), tend to be used interchangeably in the literature and in discussions among clinical staff and with families.^15,16^ The definitions underlying these terms vary across studies and publications, creating difficulty in comparing research results and confusion between professionals and families. There are concerns that previously used definitions of NE, for example that of the ACOG-AAP, do not include a clear definition covering the full spectrum of infants who may be affected by encephalopathy. They may exclude infants who do not meet the current criteria for therapeutic hypothermia, thus excluding infants with mild NE and/or under 35 weeks of gestation. While these infants may not be appropriate candidates for currently available therapies, they are at higher risk of poor neurological and developmental outcomes compared to their peers.^17,18^ Knowledge of the benefits and risks of treatment for mildly affected infants awaits focused clinical trials.

To date, many trials have assessed the neuroprotective potential of a number of different strategies. The major clinical trials in this area have assessed whole body hypothermia,^4,6,9,19^ selective head cooling,^5^ depth and duration of TH^20^ and erythropoietin.^10^ Regardless of the terminology used in these trials, which varied between encephalopathy, HIE and PA, they attempted to identify a cohort of infants with encephalopathy secondary to a purely hypoxic aetiology. This needed to be done early in the infant’s life to facilitate commencement of treatment before it would be possible to diagnose HIE by the criteria set down by ACOG-AAP. Although the criteria employed by these trials were relatively similar, there were subtle differences. Of the major trials of induced hypothermia, all except the ICE trial used a gestational threshold of 36 weeks.^21^ Trials within the NICHD neonatal research network^4,20^ and the HEAL trial^10^ mandated severe birth acidosis (pH <7.0 or BD 16 in umbilical cord gas or gas within 1 h of birth). If a blood gas was not available or was borderline, documented acute perinatal event and prolonged birth depression (either a 10-min Apgar score of 5 or less or assisted ventilation initiated at birth and continued for at least 10 min) was required. In contrast, other major hypothermia trials did not mandate severe birth acidosis. Neonates with either severe birth acidosis or prolonged birth depression were eligible for recruitment. The HELIX trial required only short birth depression (Apgar score of <5 at 5 mins or continued for at least 5 min) and did not mandate severe birth acidosis as an inclusion criterion.^9^ Further study of the HELIX trial has shown that the subgroup of infants with documented acidosis or prolonged depression (APGAR < 6 at 10 min or resuscitation requirement at 10 min), those meeting criteria set by other studies, did not have an additional neuroprotective benefit from induced hypothermia.^19^ The NICHD neonatal research network trials,^4,20^ HELIX trial^9^ and HEAL^10^ trial mandated neurological assessment by examiners certified on modified Sarnat stage and three or more abnormalities under moderate or severe category was required for eligibility. The Cool Cap trial,^5^ TOBY trial,^6^ neo.nEURO trial^22^ required only two neurological abnormalities on clinical assessment but mandated an abnormal amplitude integrated EEG as an inclusion criterion. The ICE trial did not standardise the neurological assessment nor use aEEG, and 19% of the recruited neonates had mild encephalopathy.

The early timepoint required excluded pre-randomization confirmation of other pathologies as a cause of encephalopathies, including genetic and metabolic conditions. None of these trials have reported how many infants had aberrations of growth (head circumference or birthweight for gestational age that departed from the mean by + or − 2 SD or more), were subsequently diagnosed with major malformations or genetic or metabolic disorders that may have contributed to the original presentation. Nor have existing trials reported what percentage of infants had expert examination of the placenta or the results of such examination. Up to 10% had a co-existing sepsis diagnosed which will have contributed to encephalopathic presentation but is not generally considered an exclusion criterion to these treatments. The development of a definition for the umbrella term of NE, which later can be subdivided by aetiology, while maintaining an ability to identify an infant with a presumed or likely hypoxic ischaemic aetiology, will help in the identification of all infants who may benefit from novel treatment strategies, such as those less than 36 weeks gestation, those with a mild encephalopathy, those with higher Apgar scores or less initial acidosis who may still have poor outcomes, those who present later than a 6 h cut-off point, and those with aetiologies such as stroke, genetic or metabolic conditions who, although they may not be the target population for these trial, may be included inadvertently. A broader definition will also aid in the performance of studies in aetiologies that are not purely hypoxic ischaemic in origin.

The use of terminology in this area has been controversial and this has precluded the adoption of previous definitions and diagnostic criteria widely. We believe that part of the reason for this may be because the opinions and lived experience of the wide range of people involved in the care of infants with Neonatal Encephalopathy may not have been incorporated. The involvement of a wide range of experts will aid with implementation and adoption of the final definition. The use of a Delphi consensus process rather than a data-driven process will allow for these opinions to be considered and incorporated.

Clear and internationally consistent definitions and criteria are needed to define NE and its subgroups for the next steps in research on risk factors, natural history, and treatments, and for communication of results. The aim of the DEFINE project, as outlined by the steering group of the project, an international group of experts in NE that includes parent representatives, is the development of an international multidisciplinary consensus definition, using a Delphi consensus approach.

## Methods/design

The development of this definitional statement and set of diagnostic criteria will adhere to the ACcurate COnsensus Reporting Document (ACCORD) guideline for reporting consensus-based methods without a definitive set of recommendations on forming consensus-based definitions or diagnostic criteria.^23^ We will also base our work on the methods employed by our group previously in the consensus-based development of definitions for other disease processes and core outcome sets and those outside our group.^24–26^

**Fig. 2:**
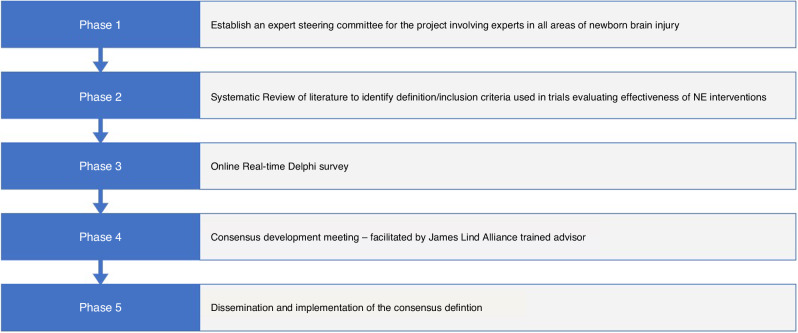
The DEFiNE project outline. An outline of the five stage process this project will proceed through to develop a consensus definition for Neonatal Encephalopathy.

There will be five phases in this work (Fig. 2).

**Phase 1**: We will establish a steering group for the project involving experts in the area—those who have an in-depth knowledge of infants who present in the early days of life with disturbed neurological function or signs of brain injury, and those who care for and follow these children and adults as they grow, including families and caregivers.

**Phase 2**: In parallel with the formation of the steering group, a systematic review of the literature will be performed to identify the definition/inclusion criteria used in the previously published literature on trials evaluating the effectiveness of interventions for managing NE.

**Phase 3**: Online Real-time Delphi survey—a number of domains, each with an included set of definitional statements, will be put forward for voting for inclusion/exclusion in the NE definition.

**Phase 4**: Consensus development meeting to agree on the final wording of an NE definition and set of diagnostic criteria – facilitated by a James Lind Alliance trained advisor.

**Phase 5**: Dissemination and implementation of the consensus definition

### Phase 1: development of steering committees

A steering committee will be formed, including international experts in this field. The steering group for DEFINE will include neonatologists, neurologists, obstetricians, metabolic paediatricians, neonatal nurses, midwifes, allied health professionals, paediatric rehabilitation specialists, parents of infants with NE and representatives from parents’ groups (i.e. public and patient involvement representatives), experts in research methodology, and clinical and scientific researchers in NE. We will endeavour to have input from both high and low-middle-income countries and a broad geographical spread. The collective knowledge of this group will inform the creation of the Delphi Study and the development of the definition/diagnostic criteria. The James Lind Alliance will be approached by the steering committee and invited to act as experts in the design and running of, and facilitators for the consensus development meeting, inspired by James Lind Alliance methods. Ethical approval for the project will be obtained before commencing from the Ethics Committee of The Coombe Hospital, Dublin, Ireland.

### Phase 2: systematic review

#### Research question: what descriptive terminology and definitions are used in clinical trials of neonatal encephalopathy

We will conduct a systematic review of randomized trials evaluating the effectiveness of interventions used for treating Neonatal Encephalopathy (NE), to identify the descriptive term(s) used to identify NE/Perinatal Asphyxia (PA)/HIE and the definition/diagnostic criteria used in each trial. This systematic review was developed as an extension of the search of a registered protocol with Prospero (CRD42020170265), a systematic review of reported outcomes in randomized control trials in NE.

##### Inclusion criteria

Types of Studies—Randomized trials evaluating the effectiveness of interventions for treating NE.

Types of Participants—Infants with a reported clinical diagnosis of and receiving treatment for NE with a gestational age greater than 35 weeks. Any definition of NE/HIE/PA that includes features of PA or encephalopathy will be acceptable.

Types of Intervention—Any intervention to treat NE or HIE. Comparison/control group may be an alternative intervention, placebo treatment or control (no treatment) group.

Types of Outcomes—Description of the terminology, definitions and diagnostic criteria used to describe term infants who are encephalopathic after birth and their frequency of use within clinical trials of interventions.

##### Search methodology

Five databases will be systematically searched: Embase, Medline (PubMed), the Cochrane Central Register of Controlled Trials (CENTRAL), Cochrane Database of Systematic Reviews (CDSR) and the World Health Organization International Clinical Trials Registry Platform (WHO-ICTRP) – ongoing trials. Searches will be restricted to the last 20 years and to articles in English. Additional references will be identified via reference searching and discussion with experts in the area.

##### Assessment for eligibility

The titles and abstracts of each citation identified by the search strategy will be screened independently by two reviewers. Full-text examination will be carried out on potentially relevant citations. Disagreements will be resolved through discussion with a third reviewer.

Papers will be eligible for inclusion if they are randomised control trials of interventions in NE/HIE/PA performed in humans. All interventions will be acceptable for inclusion. Studies of other design types (case reports, case series, in vitro studies, or animal studies) will be excluded as will studies not in the English language or performed outside of the past 20-year period.

##### Data extraction

Data will be extracted from each study into a purposefully designed data extraction form, including study design, author details, year, journal of publication, the country in which the study was conducted, the term used for the targeted condition, the definition used for this term, criteria for the diagnosis of target condition, interventions under investigation and outcomes. Data will be extracted independently by two individual reviewers. Disagreement will be resolved through discussion with a third reviewer.

##### Data analysis and presentation

Data will be tabulated using an Excel spreadsheet. No meta-analysis is proposed for this review. The terminology used will be divided into PA/NE and HIE. Data will be grouped into perinatal asphyxia and neurological assessment categories. All quantitative analyses will be conducted with IBM-SPSS Version 28 software.

### Phase 3: online Real-time Delphi survey

The Delphi method has been used widely and successfully to achieve consensus among experts since its development in the 1960s. It has been successfully employed in several areas of medicine to achieve consensus on core outcome sets and definitions, among others.^25–27^ The traditional Delphi approach uses an iterative process with repeated rounds of evaluation and voting, with feedback provided between rounds to arrive anonymously at a consensus. The Real-time method was developed to decrease the time taken and risk of participant attrition seen in traditional Delphi methods. In this round-less method, participants can view the group response in real-time after responding and revisit and re-rate responses based on group feedback.^28^ The Real-Time Delphi (RTD) approach has been successfully used in the medicine.^29,30^ A randomized trial of the traditional approach and real-time approach for developing a core outcome set in NE showed no differences in results between the two methods. The multi-round approach had larger participant drop-out, longer time to completion and less convergence of scores than the RTD approach.^31,32^

#### Design

The identified publications from the systematic review will inform the Delphi process. The definition used in each paper will be identified and the language and features used to characterize NE/HIE or PA will be extracted. Each definition will be structured into several domains (for example evidence of perinatal asphyxia, neurological assessment) based on common themes found within the identified definitions. Within each domain, we will form several statements, ideas or concepts covering different aspects within the domain and covering the breadth of opinion on what may be considered valuable for inclusion. Respondents will first vote on their opinion of the domain as a whole, before voting on the definitional statements within.

The Delphi questionnaire will be pre-piloted by a smaller working group with experience in design and conduct of Real-time Delphi (RTD) survey design and conduct, and representatives from parent and Public and/or Patient Involvement (PPI) groups to ensure accessibility of the survey to a broad audience. The steering committee will assess and approve the contents of the Delphi questionnaire before the commencement of the online process, with the potential to add domains or statements as appropriate.

#### Setting

The consensus process will take place online. Calibrum (Surveylet) software (https://calibrum.com) will be used to facilitate the real-time aspect based on the results of studies comparing software platforms.^33,34^

#### Participants and recruitment

We will recruit participants with expertise in NE from a broad range of stakeholder groups. These include healthcare providers (neonatologists, neurologists, obstetricians, midwives, neonatal nurses, allied health professionals), researchers and parents/family members/guardians/representatives of children with NE or adults who had NE as infants. We will disseminate an invitation to participate to target participants as follows: we will use electronic discussion lists, contact those who have previously participated in similar research work carried out by this group, and contact experts who have published in this area identified through the systematic review, professional organizations, and parent/family support networks and organizations.

Participants will be asked to provide informed consent after reading the participant information leaflet and before commencing the process. Before participation, they will be asked to complete a short demographic survey (stakeholder group, level of experience, country of work, ethnicity and basic demographic details).

Purposeful sampling will be used to ensure that participants representing each stakeholder group from both high and low-to-middle-income countries are recruited. We will ensure the participation of each group of stakeholders at each stage of the consensus process. Participants will be grouped broadly into three groups: (a) parents/family members/guardians of infants who received care for NE, (b) healthcare providers, and (c) researchers and policymakers. The survey will be live for 4 months, and we will aim to recruit as many participants as possible within each stakeholder group. We will aim for equal representation across stakeholder groups. However, acknowledging that the technical nature of the survey may make it difficult for non-medical participants, if one group is lower than expected we will endeavour to support participation in the survey itself and in the later consensus meeting to ensure their views are well represented.

In the RTD participants will be asked to rate the level of importance they attach to potential components to include or exclude in a definition of NE. They will do so for each domain initially and then each statement within that domain using a 5-point Likert scale (strongly agree with inclusion/agree with inclusion/need more information or clarification/disagree with inclusion/strongly disagree with inclusion).

For each domain and statement, the participant can view their rating of the item, the overall rating of the item and the rating of the item for each stakeholder group immediately after rating the item for the first time. After viewing the feedback, the participant can, if they choose, modify their response before moving to the next item.

A free-text box will be provided alongside each domain/statement that the respondent is asked to vote on. The participant can use this to add additional detail they feel important to include, clarify reasoning, or propose alternative wording if they agree with the premise of the statement overall but not the exact wording. The participants will be able to view the comments made by other participants in real-time, identified by stakeholder group – as it may benefit other participants to understand the area of expertise of those providing comments. If participants would prefer to remain anonymous only stakeholder group will be reported. Participants will also be able to suggest additional statements that they feel should be added to each domain.

Participants can save their responses and revisit them before the pre-determined completion date. As the degree of consensus changes, depending on each additional rating, we will encourage participants via email reminders to revisit the survey and review their answers. No new participants will be recruited the week before the completion date to ensure adequate time for engagement with the process. The steering group will decide the timing and frequency of email reminders based on temporal responses and level of engagement.

The order in which the domains are presented to participants will be randomized to decrease the influence of order on question responses^35^ [17]. Medical terminology will be used in each statement where relevant, with a plain-language summary provided to participants. To avoid a small group of initial participants having a significant influence on consensus levels and potentially biasing early participants, a representative group from each stakeholder group, and members of the steering committee, will complete the real-time Delphi survey before it goes live more broadly. In this way, consensus information will be provided to each online participant in the same manner.

An 80% consensus threshold will be used for the inclusion and exclusion of statements in the final definition. If there is agreement by 80% of participants overall on a statement (i.e., 80% of participants vote strongly agree or agree with inclusion) it will then be included/excluded. An 80% threshold was used based on previous similar work and evidence from the literature that suggests 80% agreement is needed for validity in consensus in groups of greater than ten experts.^36^

The steering group will follow the progress of the RTD process. If necessary, the process will be paused to allow for analysis of responses, clarification of statements or addition of statements suggested by participants and removal of those reaching consensus. After pausing to allow this, the process will be recommenced for participants as outlined above. If several potentially contradictory statements are identified, these will be grouped, and participants will be asked to vote on their level of agreement with each statement.

### Phase 4: consensus meeting

#### Objective

The aim of the consensus meeting will be to achieve a final agreement on the wording of one unifying definition for the term NE, and a set of diagnostic criteria for NE, based on the findings of the Delphi consensus process through online meetings of international stakeholders with expertise in NE.

We will hold at least two independent consensus meetings with different stakeholders, including at least three people from each stakeholder group (clinicians, parent and patient representatives, scientists and policy-makers).

During the consensus meetings, all statements from the real-time Delphi will be presented to participants along with the voting patterns of the stakeholder groups. An experienced facilitator will chair each consensus meeting. There will be anonymous computerised voting after a discussion on items which did not reach a clear consensus or which had borderline agreement, using an 80% threshold on a three-point scale (critical, important, not important). The domains and statements will be formulated into the final definition, the exact wording of which will be discussed and agreed upon at the consensus meeting. The James Lind Alliance will be involved in designing and running the consensus development meetings. The James Lind Alliance primarily supports research Priority Setting Partnerships and has experience in the running of consensus processes bringing together patients, carers and clinicians. A James Lind Alliance trained Adviser will ensure all stakeholder groups are involved equally in the consensus meeting (https://www.jla.nihr.ac.uk/).

### Phase 5: dissemination and implementation strategy

Our dissemination and implementation plan will be guided by the principles put forward by many Health Research Board’s knowledge transfer strategy^37^ i.e., (i) Monitor; (ii) In-form; (iii) Knowledge Exchange; (iv) Persuade; (v) Network and (vi) Support. The outcome of our consensus process, including the definition itself and set of diagnostic criteria, will be published in an international peer-reviewed open-access journal. We will present our definition at international meetings of experts in this field and disseminate our findings via other channels, including clinical trial networks and units, and research funding agencies.

## Discussion

NE describes a disturbance of neurological function of cerebral origin in the term, or near term, newborn which does not specific the underlying aetiology. Several organizations have advocated for the use of the term NE, followed by the application of a more precise causative diagnosis, be that HIE or another, after thorough aetiological investigations have been performed.^2^ The terms NE, HIE and PA have been used interchangeably and sometimes without definition, in the literature to convey an infant with a brain injury from birth. This leads to difficulty in identifying the group of patients enroled in a clinical trial and therefore the potential generalisability of the findings and meta-analysis of the results given the heterogeneity of the primary literature. As the search for adjunctive treatments to therapeutic hypothermia, or indeed stand-alone therapies for use in low-middle income countries, continues the use of a standard term with a standard definition may aid the development of these trials and implementation of their results. For parents and caregivers of infants with NE, using consistent terminology and meaning for these terms will aid with understanding, aetiological diagnosis and access to the correct support available to them. The Delphi process has been used previously to provide consensus among large groups of experts. The modified RTD approach has been successfully employed to develop consensus.^32,38^ This protocol aims to provide a consensus-based definition and set of diagnostic criteria for the term NE, which will be accepted and utilised by the neonatal community to improve research, outcomes, and parental experience.

### Project status

Currently, this project is moving through phases 1–3 as outlined in the above protocol. The systematic review of phase 1 is being analysed and finalized. A steering group has been formed, and preliminary meetings have been held to define the scope of our process and finalize this protocol.

## Data Availability

Data can be made available on reasonable request to the corresponding author.
